# Intrinsic ankle stiffness during standing increases with ankle torque and passive stretch of the Achilles tendon

**DOI:** 10.1371/journal.pone.0193850

**Published:** 2018-03-20

**Authors:** Tania E. Sakanaka, Jaspret Gill, Martin D. Lakie, Raymond F. Reynolds

**Affiliations:** School of Sport, Exercise & Rehabilitation Sciences, University of Birmingham, Birmingham, West Midlands, United Kingodm; University of L'Aquila, ITALY

## Abstract

Individuals may stand with a range of ankle angles. Furthermore, shoes or floor surfaces may elevate or depress their heels. Here we ask how these situations impact ankle stiffness and balance. We performed two studies (each with 10 participants) in which the triceps surae, Achilles tendon and aponeurosis were stretched either passively, by rotating the support surface, or actively by leaning forward. Participants stood freely on footplates which could rotate around the ankle joint axis. Brief, small stiffness-measuring perturbations (<0.7 deg; 140 ms) were applied at intervals of 4–5 s. In study 1, participants stood at selected angles of forward lean. In study 2, normal standing was compared with passive dorsiflexion induced by 15 deg toes-up tilt of the support surface. Smaller perturbations produced higher stiffness estimates, but for all perturbation sizes stiffness increased with active torque or passive stretch. Sway was minimally affected by stretch or lean, suggesting that this did not underlie the alterations in stiffness. In quiet stance, maximum ankle stiffness is limited by the tendon. As tendon strain increases, it becomes stiffer, causing an increase in overall ankle stiffness, which would explain the effects of leaning. However, stiffness also increased considerably with passive stretch, despite a modest torque increase. We discuss possible explanations for this increase.

## Introduction

In quiet standing the ankles play a crucial role in connecting the long, nearly vertical body to the feet, which are used to apply gravitational counteractive torque against the ground. This torque is used to stabilize the inherently unstable body. Ankle torque is produced by passive and active mechanisms. The passive mechanism consists of the visco-elastic forces produced by the stretch of the muscles, tendons and ligaments acting around the ankle, and this operates with zero delay. This is the intrinsic ankle stiffness. The active mechanism is the modulation of ankle muscle activity by the nervous system. Responses to unforeseeable disturbances will be delayed, but usually in quiet standing neural prediction minimizes the delay [1]. Intrinsic ankle stiffness is not normally sufficient to stabilize the body by itself [2,3]. However, by providing passive instantaneous resistance to falling, it supplements actively generated torque and increases the time constant of the unstable body, giving more time for neural intervention [4].

Here we investigate intrinsic ankle stiffness for anterior-posterior sway (i.e. in the sagittal plane). The two main contributors to intrinsic ankle stiffness are the Achilles tendon and the triceps surae muscles, which act as springs arranged in series. During quiet standing, the stretch sizes are normally very small and ankle torque is relatively low [2]. Ankle stiffness is therefore determined by the combination of a very long and compliant tendon and nearly stationary muscles with short fibers [5]. In normal standing, the muscle is typically ~ 15 times stiffer than the tendon [6,7]. As it is the weaker link, in this condition the tendon sets the maximal value of ankle stiffness. Consequently, muscle stiffness only becomes relevant to ankle stiffness if tendon stiffness increases, as it does under conditions of high levels of torque (e.g. walking, running or jumping).

The muscle can be stiffened either by the active contraction of its fibres or by passive means. Passive means include changes in muscle length through stretch and altering the immediate history of muscle movement (e.g. leaving the muscle still for 5–10 s), which can produce thixotropic increases in stiffness [5,7–17].

Tendon stiffness is dependent on the linkage between the collagen molecules, cross-linked end-to-end within a fibril [18]. This is a completely passive mechanism. This ‘netted’ distribution, often compared to the behaviour of a knitted sock which stiffens as it is stretched, makes it possible for the tendon to be lengthened and to support high loads of tension without rupturing. Its stiffness is defined by the amount of deformation of its fibers, ranging from a slack region, when the fibers are crimped, to a linear region, when the fibers are relatively parallel, and finally microscopic and macroscopic failure regions, when the fibers start to snap, leading to rupture [19]. Thus the tendon stiffness increases with the tension that it transmits [19–27]. *In vivo* measurement of stiffness of whole bundles of tendons and muscles acting in combination was much facilitated with the introduction of the ultrasound technique [28]. For the measurement of the Achilles tendon stiffness, for example, ultrasound probes are used to track the change in position of the distal myotendinous junction of the muscle and the insertion point of the Achilles tendon. Tendon stiffness is then expressed as the slope between this change in tendon length and tendon force [28–33].

The function of the Achilles tendon is to connect the triceps surae muscles to the calcaneous bone. Therefore, it can be elongated in two different ways; either by shortening of the calf muscle fibers connected to it through active muscle contraction, or by passive dorsiflexion of the ankles. Both modes of elongation can act to increase tendon stiffness. This implies that, in standing, ankle stiffness should increase as ankle torque increases. Ankle torque can be increased by leaning forward or by dorsiflexion of the foot. However, it is important to note that in standing the underlying ankle torque is directly proportional to the angle of inclination of the body so that ankle torque cannot be arbitrarily altered without changing body lean. A further possible way of increasing ankle stiffness may be by co-contraction, although co-contraction is unusual in standing.

Two contradictory results for ankle stiffness have been reported for upright human subjects. Loram & Lakie [2] applied brief and very small (0.05 deg amplitude, 140 ms duration, squared-sine shaped) perturbations to individuals strapped to a vertical support while standing on footplates. At this fixed ankle angle, the participants were asked to maintain a constant mean level of bias plantarflexing ankle torque for 40 s. During this task mean ankle angle did not change, therefore, the tendon stiffness could only be altered by the contracting muscles pulling the tendon. Over a relatively large range of plantarflexing ankle torque (5–25 Nm in one leg only), the researchers found only a small and insignificant rise in ankle stiffness (from ~5 to ~6 Nm deg^-1^). In freely standing individuals, mean plantarflexing ankle torque can only be increased by leaning forwards. Casadio et al. [34] applied larger perturbations (1 deg, 150 ms, ramps) to freely standing individuals. They studied only two subjects, but in both there was a substantial increase in ankle stiffness as the subjects leaned forward. Because the subjects were leaning forward, there was an inevitable slight dorsiflexion.

In an attempt to resolve these conflicting observations, we carried out two experiments to further investigate how intrinsic standing stiffness can be affected by ankle torque or angle. In study 1, intrinsic stiffness was measured at various mean levels of forward lean using a range of perturbation amplitudes. In study 2, intrinsic stiffness was measured while standing normally with increased ankle dorsiflexion (that is, standing with toes raised).

The intention was to compare the effects of two conditions in which tendon tension was varied. In one it was varied mainly by muscle activation (forward body leaning) and in the other it was varied mainly by passive stretch (ankle dorsiflexion). Of course, active and passive sources of ankle torque are inevitably entangled during quiet stance, but our objective here was to maximize the contribution of either source using these two interventions. We were also able to investigate whether changes in stiffness were critically dependent on the perturbation amplitude used to make the measurement. In addition, we investigated how sway size and velocity were altered by leaning and dorsiflexion. To our knowledge, no previous study has investigated the effect of Achilles tendon tension on postural sway.

## Methods

### Participants

For each study, 10 healthy volunteers (Study 1: six female; age 28.1±4.4 years (mean±SD); height 1.68±0.1 m; weight 65.9±8.3 kg) (Study 2: six female; age 29.1±10.5 years) gave written informed consent and participated in this study, which was approved by the local human ethics committee at the University of Birmingham (Table 1).

**Table 1:** Participant anthropometric data.

Study 1			
**Participant**	Gender	Age (years)	Toppling torque per unit angle (Nm deg^-1^)
P01	M	31	8.47
P02	F	26	10.58
P03	F	37	8.26
P04	F	30	8.99
P05	F	22	8.92
P06	F	24	9.15
P07	M	31	13.27
P08	M	24	12.58
P09	M	29	9.81
P10	F	27	9.74
**Mean ± SD**	**F(6)**	**28.19±4.4**	**9.98±1.7**
**Study 2**			
**Participant**	Gender	Age (years)	Toppling torque per unit angle (Nm deg^-1^)
P01	M	39	12.42
P02	F	25	12.25
P03	F	37	7.44
P04	F	23	10.13
P05	F	23	14.76
P06	F	22	8.84
P07	M	25	9.92
P08	F	23	8.06
P09	M	53	14.97
P10	M	21	17.31
**Mean ± SD**	**F(6)**	**29.1±10.5**	**11.6±3.3**

### Procedure and apparatus

**Fig 1:**
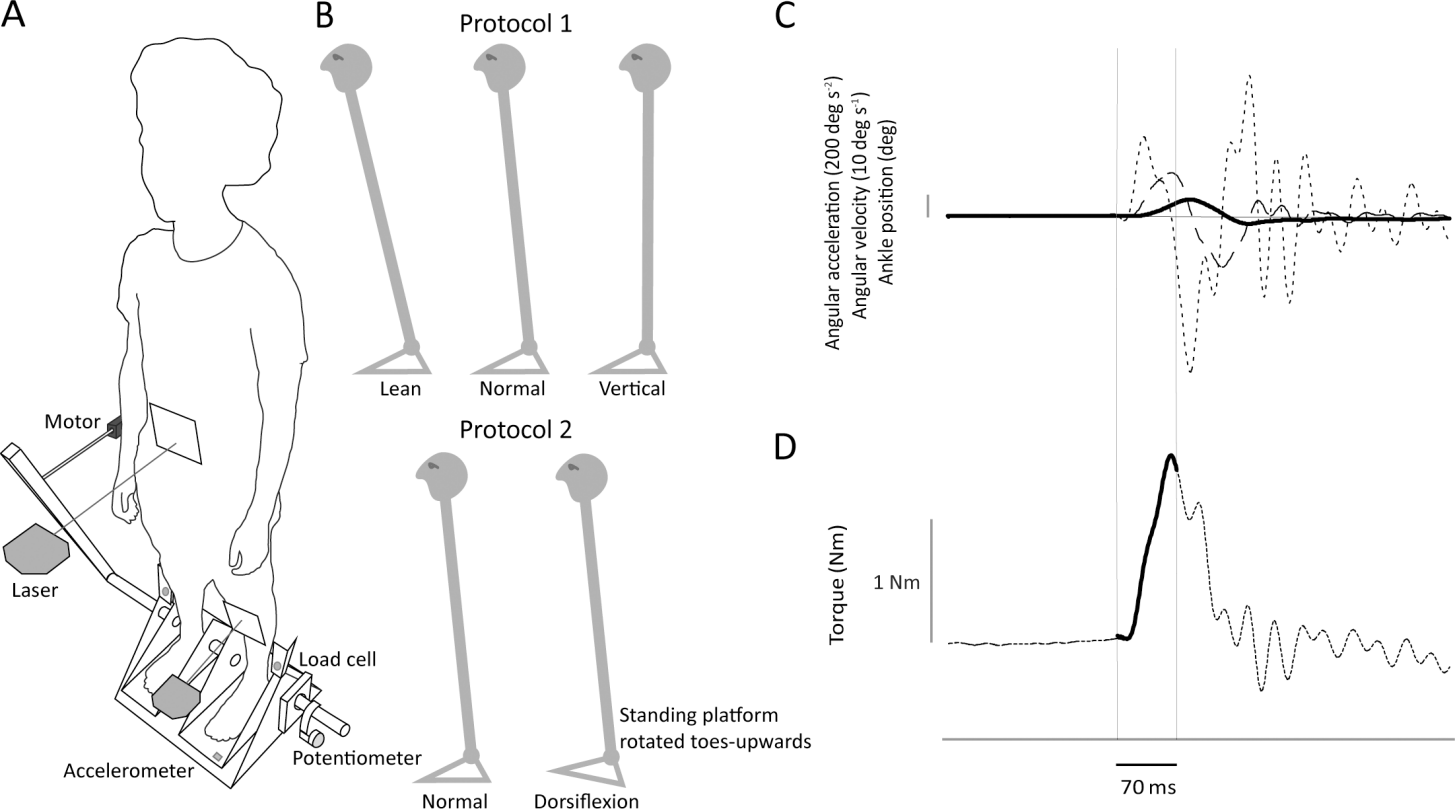
Experimental setup. (A) The position servo motor was installed horizontally and applied perturbations to the crank, thus rotating the platform and footplates. Separate load cells measured torque for each ankle. They transmitted all forces between the platform and footplates, directly above the axis of rotation. A potentiometer attached to the axis of rotation measured anteroposterior rotation of the footplate. An accelerometer attached underneath the left footplate measured its acceleration. Two laser-reflex sensors placed at left mid-tibia and umbilicus level tracked the anteroposterior shin and body tilt. (B) During study 1 (top figures), the standing platform was level and the participant altered body position. During study 2 (bottom figures), the standing platform was rotated upwards by 15 deg during the dorsiflexion condition. Only left lower limb recordings were used for stiffness and sway analysis, and surface EMG was recorded from the medial gastrocnemius and tibialis anterior muscles. (C) Example of averaged ankle angle (continuous line), angular velocity (dashed line) and angular acceleration (dotted line) data used to estimate mechanical intrinsic ankle stiffness. The time-window (70 ms) used for the analysis are indicated by the thin vertical lines. The starting point coincides with the stimulus onset. (D) Ankle torque response (dotted line) and, on top of it, reconstructed torque (continuous line) obtained from the second order model used to estimate stiffness. The bottom horizontal line indicates 14.5 Nm.

A full description of the footplate apparatus used to measure ankle stiffness was given elsewhere [5]. In brief, the participants were asked to stand on top of motorized footplates, coaxially aligned with their ankles. Ankle torque and angle and left footplate acceleration were recorded, as well as lower limb EMG responses from the *medial gastrocnemius* and *tibialis anterior* muscles (Delsys Bagnoli). Only measurements of the left lower limb were used for the stiffness estimation. The methodology specific to the two studies is described below (Fig 1).

We chose to record medial gastrocnemius rather than soleus activity because responses due to reflex or higher level activity are more prominent and easier to identify in that muscle [2,35,36]. However, previous research reports cross-talk between the triceps surae muscles when using surface EMG [37], so it is possible that the recording is representative of activity in the entire calf muscle. For clarification, we suggest recording the activity of both medial gastrocnemius and soleus muscles in future studies.

#### Study 1

The main objective of this experiment was to determine how passive ankle stiffness changes with increasing ankle torque caused by forward lean. Participants stood for approximately 3 minutes, during which small and brief perturbations were applied at a variable gap of 4–5 seconds. Each perturbation was a raised cosine curve of 140 ms duration with a randomly varied maximum amplitude of 0.1, 0.3 or 0.7 deg, in a randomly varied toes-up and toes-down direction. The three amplitudes were chosen to establish whether the effect of forward lean on ankle stiffness was critically dependent on the amplitude of the perturbation used to measure it. The strong perturbation repeatability was confirmed when calculating the standard deviation of its peak angular rotation (potentiometer recordings). Within the data of a single participant, it varied by as little as 0.013 deg SD for 0.1 deg perturbation, 0.013 deg SD for 0.3 deg perturbation and 0.017 deg SD for 0.7 deg perturbation. The whole experimental procedure consisted of one session of approximately 1 ½ hours.

We asked the participants to manipulate the amount of forward shift of their COP by monitoring the average baseline torque applied by the feet against the footplate. Before the experiment, participants did at least one familiarization trial per condition to establish the level of torque that they could sustain at each level of leaning. Due to individual variation of weight and height, it was not possible to establish a common target torque for all participants. For example, during the forward leaning condition, we asked the participants to lean as far forward as they could, comfortably for the duration of the trial. We averaged these torque traces and established this value as an individual target trace they had to maintain. It was displayed on a screen located at eye level during the actual trials. They performed three different levels of forward leaning:

1. *Normal*: standing at their spontaneously chosen position;
2. *Vertical*: standing with the COP shifted backwards in relation to their normal condition. Participants were asked to reduce torque applied against the footplate, as much as possible without compromising their free standing balance control;
3. *Lean*: standing with the COP shifted forwards in relation to their normal condition. Participants were asked to increase torque applied against the footplate to a level that was still comfortable and sustainable for the duration of the 3 min trials.

For obvious reasons, it was relatively easier for individuals to follow a target torque in normal condition (0.67±0.53 Nm, mean±SD, difference from target torque). It was more difficult to maintain proximity with the target torque in vertical and lean conditions (1.02±0.92 Nm and 1.91±1.69 Nm, respectively). Nevertheless, the participants were able to maintain a reasonable amount of accuracy, considering that they were freely standing.

There were 3 conditions of COP shift (normal, vertical and lean) and 3 different perturbation amplitudes (0.1, 0.3 and 0.7 deg), resulting in a total of 9 conditions. Given that 30 perturbations were applied per condition, altogether 270 events were recorded for each participant. The timing, conditions, perturbation amplitudes and directions were pseudo-randomized so that they could not be predicted by the participants.

#### Study 2

For this experiment the main objective was to increase ankle dorsiflexion with minimal change in baseline ankle torque. Participants performed standing trials with perturbations of the same shape and time-window intervals as in the previous experiment, but only with amplitudes of 0.1 or 0.7 deg. There were two different conditions:

1. *Normal*: standing at their spontaneously chosen position with footplates horizontal;
2. *Dorsiflexion*: standing with the footplate rotated upwards by 15 deg. As it was an angular measure, it was common to all subjects (unlike the torque in study 1). 15 deg was chosen because it was close to the maximum that the participants could comfortably maintain.

The whole experimental procedure consisted of one session of approximately 1 hour. There were 2 ankle positions and 2 perturbation amplitudes and 32 perturbations were recorded for each condition, resulting in a total of 128 events from each participant. Normal standing was investigated prior to the dorsiflexion trials. Perturbation amplitudes and directions were pseudo-randomized.

### Data analysis

#### Determination of mechanical intrinsic ankle stiffness

We assumed that the calf muscles, the Achilles tendon, aponeurosis and foot act as a mass-spring-damper system [38,39] responsible for generating the corrective torque applied by the feet against the ground to stabilize position [40,41]. The spring component (combination of muscles, tendon, aponeurosis and foot modulating stiffness of the ankles), damper component (viscosity of the ankle joint and associated tissues) and mass component (moment of inertia of the foot and moving muscle with respect to the medial malleolus acting as the axis of rotation) were estimated with a fitting equation in which the torque measured over the first 70 ms of the perturbation was compared with the torque generated by a simple second-order model. The three inputs to this model were the measured ankle position, velocity and acceleration [2,42]:

$$T=K\theta +B\dot{\theta }+I\ddot{\theta }$$ (1)

Where: T = torque (Nm), *θ* = angle (deg) $\dot{\theta }$ = angular velocity (deg s^-1^), $\ddot{\theta }$ = angular acceleration (deg s^-2^), K = stiffness (Nm deg^-1^), B = viscosity (Nm s deg^-1^) and I = moment of inertia of the foot (kg m^2^).

#### Determination of toppling torque per unit angle

During normal standing, the gravitational torque exerted by the body COM is closely related to the COM rotation around the ankle joint [40,43,44]. This is because the body above the ankles is kept relatively aligned in the vertical position and its sway amplitude is below 6 deg [45]. Toppling torque per unit angle is a representation of this relationship. It is defined as m × g × h (for convenience, referred as ´mgh´), where m is the participant mass above the ankles, g is the gravitational acceleration and h is the height of the COM above the ankles. Here it is used only as a reference to normalize data from all participants, regardless of their height and body mass. The intrinsic ankle stiffness was then estimated as a percentage of mgh–if equal or higher, it would potentially stabilize the body alone. Mgh was indirectly calculated as the slope of the linear fit between ankle torque (load cell data) and body angle (umbilical laser-reflex sensor data), recorded during 180 s trials of voluntary sway, in which subjects were instructed to sway very gently about the ankle joint, minimizing any hip or knee motion.

#### Determination of baseline ankle torque, ankle and body angle, body sway and EMG activity

We assessed the amount of leaning as the increase in total ankle torque. This was calculated from the mean total ankle torque during a 70 ms time window prior to each perturbation onset. The amount of ankle dorsiflexion and body inclination were calculated as the mean ankle angle (calculated from laser signal reflecting shin minus footplate angle) and body angle (laser signal reflecting movement of the waist) over a 2 s time window prior to each perturbation onset. We normalized each participant’s data to their normal standing condition, described here as 0 deg for body angle and 90 deg for ankle angle. Thus we discounted their actual elected standing position, which corresponds to a variable 1.5–4 deg forward leaning in relation to the earth [2].

The effect of the different conditions upon stability and control of movement was assessed with measurements of anterior-posterior body sway and muscle activity. Body sway was quantified as the average root-mean-square (RMS) ankle angle and velocity. The mean value over a 2 s time window prior to each perturbation onset was subtracted. Muscle activity was calculated as the integral of the rectified EMG activity of the medial gastrocnemius and tibialis anterior muscles over a 70 ms time window envelope prior to each perturbation onset. To compare changes within conditions, we normalized all participants’ EMG data as a ratio of normal standing data.

#### Statistical analysis

Repeated-measures ANOVA was used to determine effects of condition (normal, vertical and lean or normal and dorsiflexion, for 1^st^ and 2^nd^ studies) and stimulus amplitude (0.1, 0.3 and 0.7 deg or 0.1 and 0.7 deg, respectively) upon ankle stiffness. Two-tailed paired samples t-tests and one-way ANOVA were used to verify differences in mean ankle and body angle, ankle torque, body sway and EMG activity between conditions. P<0.05 was considered statistically significant for all tests.

## Results

**Fig 2:**
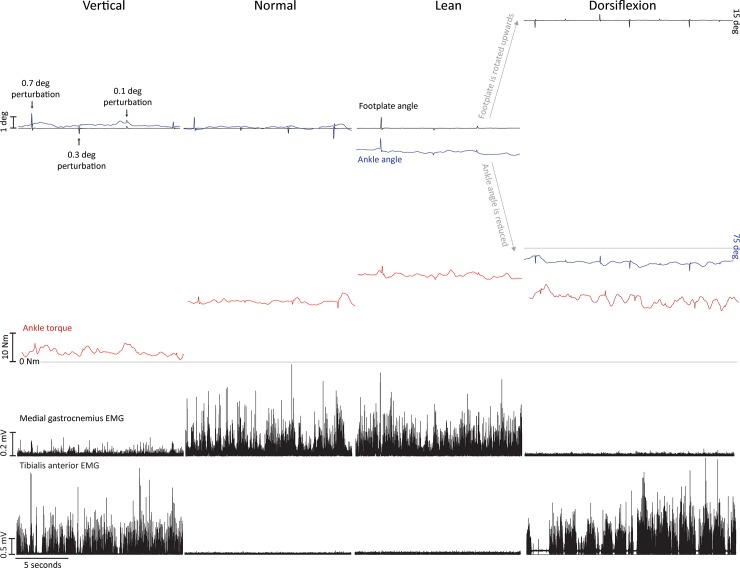
Representative data. Effects of active ankle torque and passive tendon stretch on ankle angle (footplate minus shin angle), left ankle torque, rectified left medial gastrocnemius and tibialis anterior EMG. Top panel are data from study 1 and bottom panel are data from study 2, all taken from one participant. The horizontal line beneath the torque traces represents 0 Nm. For study 2, ankle angle equals 90 deg when the footplate is levelled; it decreases (in this case to ~ 73 deg) when the footplate rotates upwards from 0 deg to 15 deg. The difference (from 75 deg) is due to body and leg movement associated with the toes-up stance.

Representative data of one participant during two different studies is shown in Fig 2. The upper panel is from study 1, while the lower panel is from study 2.

### Absolute ankle torque and relative ankle and body position

**Fig 3:**
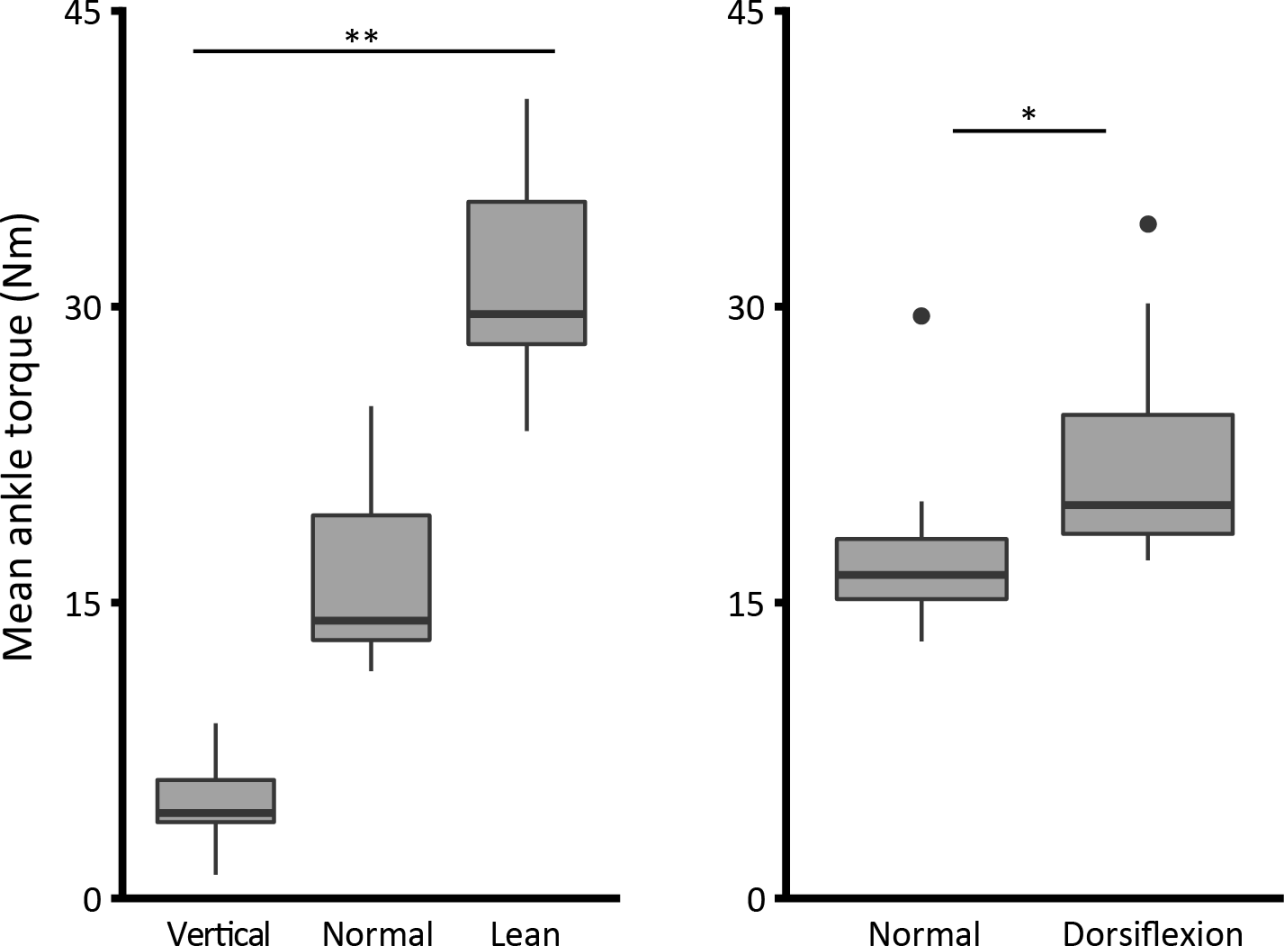
Mean ankle torque (Nm). (*) indicates significance of P<0.05, and (**) indicates P<0.001. This and the following box plots show first (bottom), second (band inside the box) and third (top) quartiles; whiskers show 1.5 IQR (Tukey box plot).

As intended, there was a significant increase in mean ankle torque between each of the conditions within study 1, ranging from 4.9±2.4 Nm (*vertical*, mean±SD), 16.4±4.7 Nm (*normal*) and 31±5.7 Nm (*lean*). A one-way ANOVA revealed a significant difference between different leaning conditions (F_2,27_ = 85.5; p<0.001) (Fig 3, left graph). In study 2, our main concern was to change the amount of ankle dorsiflexion by means of tilting the standing platform by 15 deg. There was an unintended but significant torque increase from 17.6±4.7 to 22.4±5.7 Nm (t_(9)_ = -2.4; p<0.05) (Fig 3, right graph), a result of the participants leaning slightly more forwards when the toes were raised.

**Fig 4:**
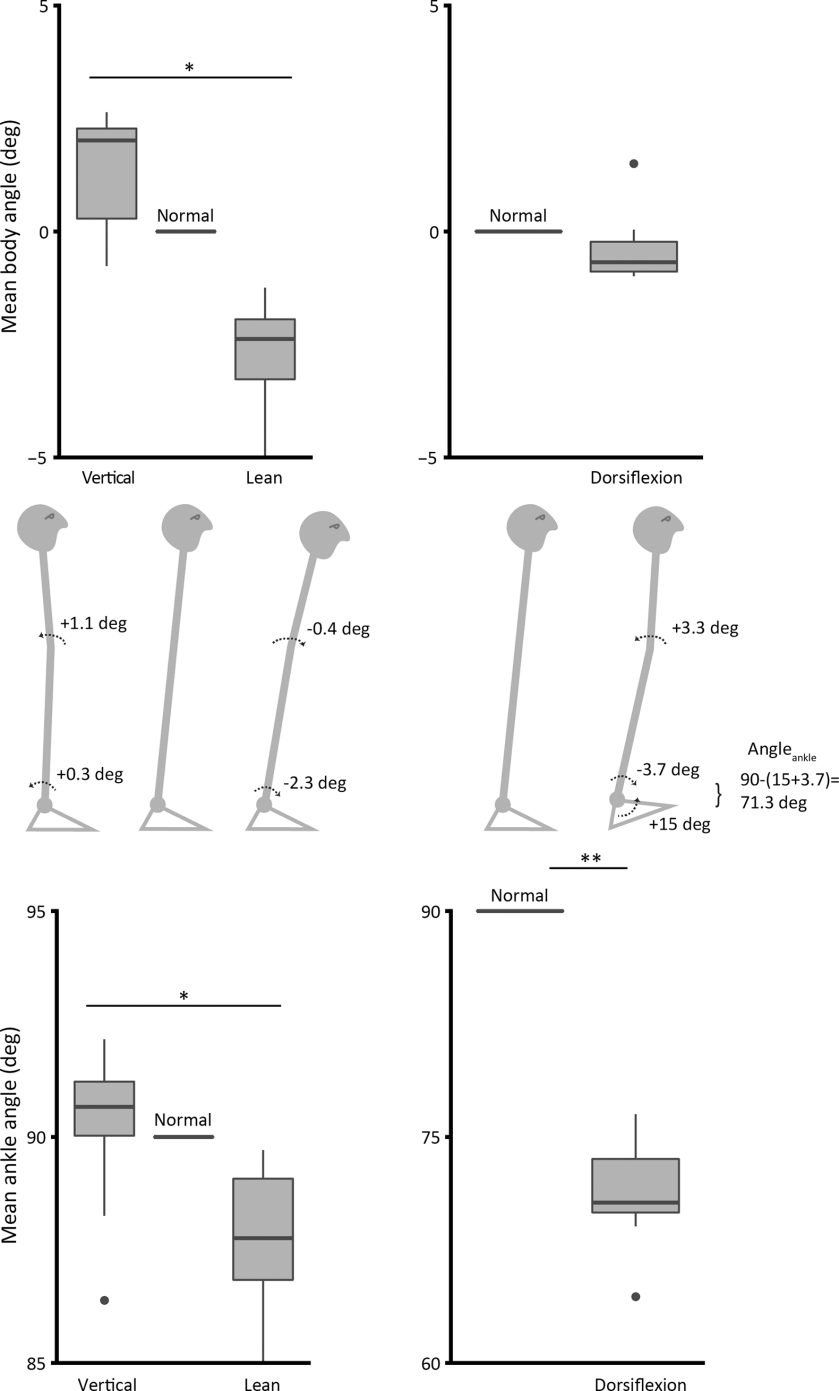
**Mean body (top) and ankle (bottom) angle (deg) relative to normal condition (= 0 for body angle, = 90 for ankle angle).** Schematic representation of the relative change in body and ankle angle is shown in the middle panel. In all conditions (particularly the toes-up condition) the body changes its postural configuration. (*) indicates significance of P<0.05, and (**) indicates P<0.001.

We next describe the changes in ankle and body angle in each experiment. In study 1, both ankle and body angle increased as the participants leaned forward. However, they did not increase by the same amount and it was clear that the subjects did not act entirely as a rigid body (Fig 4). Nevertheless, one-way ANOVA analysis has shown significant difference between conditions in body (F_2,27_ = 5.9; p = 0.007) and ankle (F_2,27_ = 3.5; p = 0.043) mean angles (Fig 4, left graphs).

Rotating the platform toes-up by 15 deg was enough to produce a large increase in ankle dorsiflexion in study 2 (t_(9)_ = -17.6; p<0.001) (Fig 4, bottom right graph). With the analysis of the body average position, we verified that even though instructed otherwise, the participants leaned forward by a slight amount (0.4±0.7 deg), but this was not significant (t_(9)_ = -1.7; p = 0.12) (Fig 4, top right graph). In leaning forward there was considerable departure from a rigid body with the body arching (convex anteriorly) so that ankle angle moved forward by 3.7 deg and body angle by only 0.4 deg. An imposed dorsiflexion of 15 deg combined with an unintentional forward rotation of the ankle of 3.7 deg generated a shift in ankle angle from the nominal 90 deg to a mean of 71.3 deg.

### Intrinsic ankle stiffness

**Fig 5:**
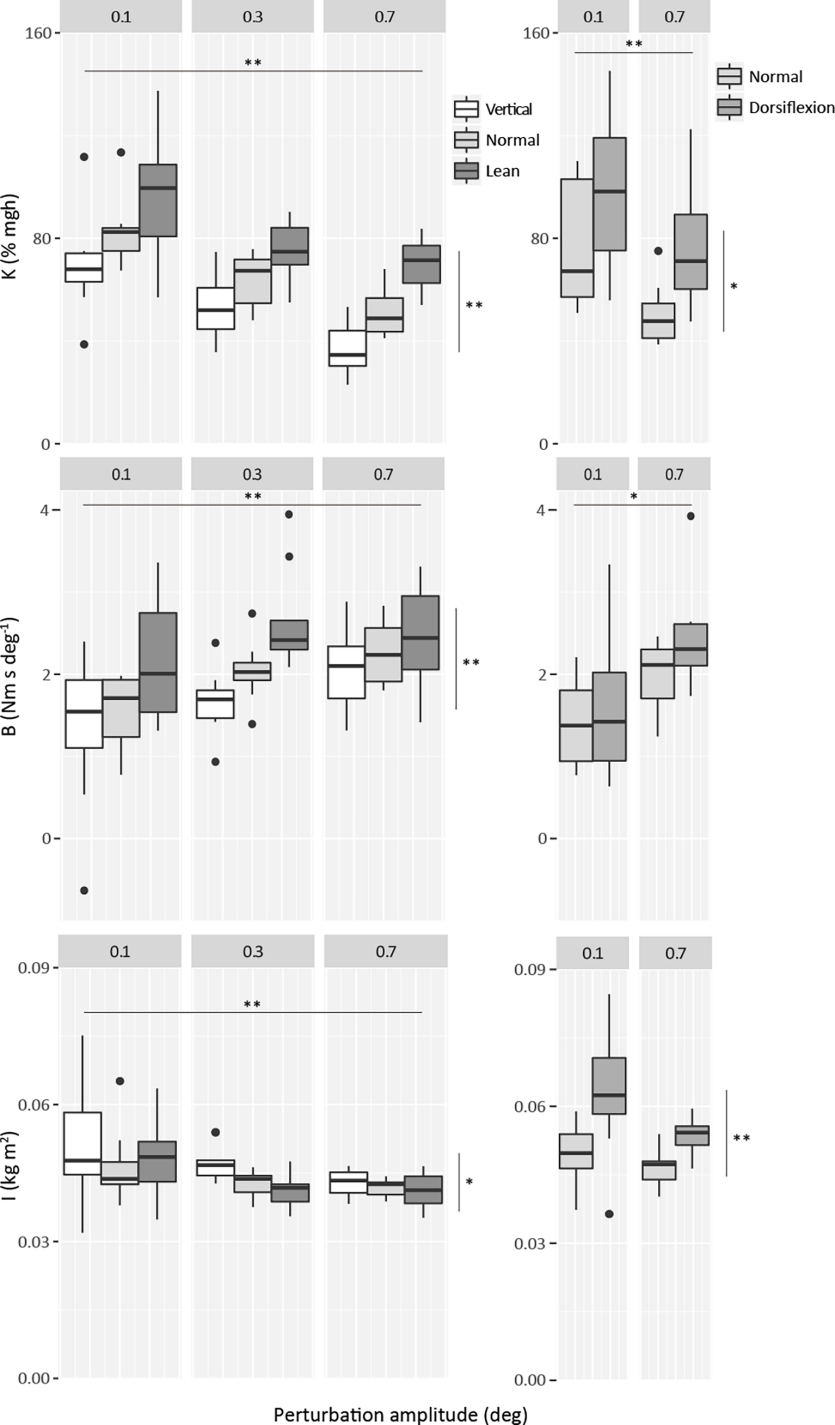
Intrinsic standing ankle stiffness (% mgh), viscosity (Nm s deg^-1^) and moment of inertia (kg m^2^) against perturbation amplitude (deg). Intrinsic stiffness was estimated with the left torque values multiplied by 2. Viscosity and moment of inertia values are from left ankle only. Horizontal lines indicate significance of perturbation amplitude, whereas vertical lines indicate significance of condition. (*) indicates significance of P<0.05, and (**) indicates P<0.001.

For both studies, average intrinsic ankle stiffness is presented as a percentage of toppling torque per unit angle (% mgh) in Fig 5. A 3-way ANOVA analysis showed that there was no effect of perturbation direction (toes-up *versus* toes-down) on stiffness, for either study (F1,9≤0.37; p = ≥0.56). Therefore, we combined perturbations of both directions for the final calculation. For the first experiment (left graph), values ranged from 37% to 97%. There was a systematic increase in stiffness (approximately 30% overall) from vertical to normal and from normal to lean (effect of condition: F_2,18_ = 18.5; p<0.001; effect of amplitude: F_2,18_ = 170.2; p<0.001). There was no interaction between condition and amplitude (F_4,36_ = 0.63; p = 0.64). Ankle dorsiflexion also produced a significant increase in stiffness (mean 29%) (condition: F_1,9_ = 18.4; p = 0.002; amplitude: F_1,9_ = 40.2; p<0.001), also without any interaction between condition and amplitude (F_1,9_ = 0.31; p = 0.59) (right graph). The results obtained from the normal condition in both experiments were very similar (51% - 50% for 0.7 deg perturbation and 82% - 77% for 0.1 deg perturbation, respectively), making comparisons between both experiments easier to interpret.

As the duration of all perturbation sizes was the same (140 ms), the peak velocity was higher for larger perturbations, and we found a significant increase in viscosity with perturbation amplitude (first study: F_2,18_ = 13.4; p<0.001; second study: F_1,9_ = 12.8; p = 0.006). Viscosity was also significantly affected by the degree of forward leaning (F_2,18_ = 13.7; p<0.001) but not by passive stretch (F_1,9_ = 2.1; p = 0.177). Moment of inertia was dependent on degree of forward leaning and amplitude in the first study (condition: F_2,18_ = 4.0; p = 0.037; amplitude: F_2,18_ = 10.6; p = 0.001); it was dependent on passive stretch but not amplitude in the second study (condition: F_1,9_ = 42.4; p<0.001; amplitude: F_1,9_ = 1.6; p = 0.233).

### Body sway and muscle activity

**Fig 6:**
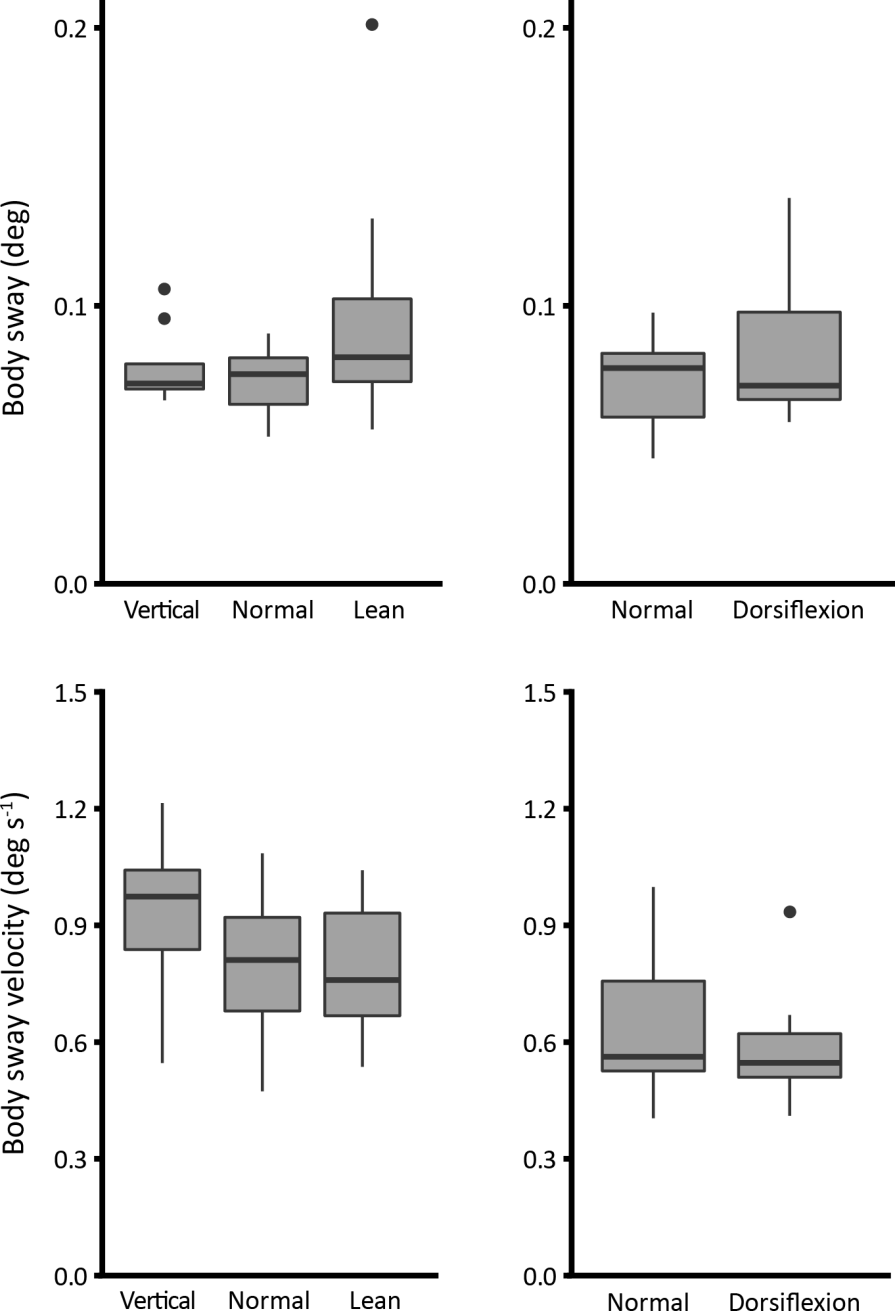
Body sway size (deg) and sway velocity (deg s^-1^).

Body sway size is described in Fig 6. Assessment of sway (body angle RMS, top left panel) and sway velocity (body angular velocity RMS, bottom left panel) show no significant difference between conditions of forward leaning (one-way ANOVA F_2,27_ = 2.2; p = 0.133; F_2,27_ = 1.8; p = 0.189, respectively). Dorsiflexion also has no significant effect on sway size or velocity (t_(9)_ = -1.3; p = 0.225 and t_(9)_ = 1.2; p = 0.267, respectively) (right panels).

**Fig 7:**
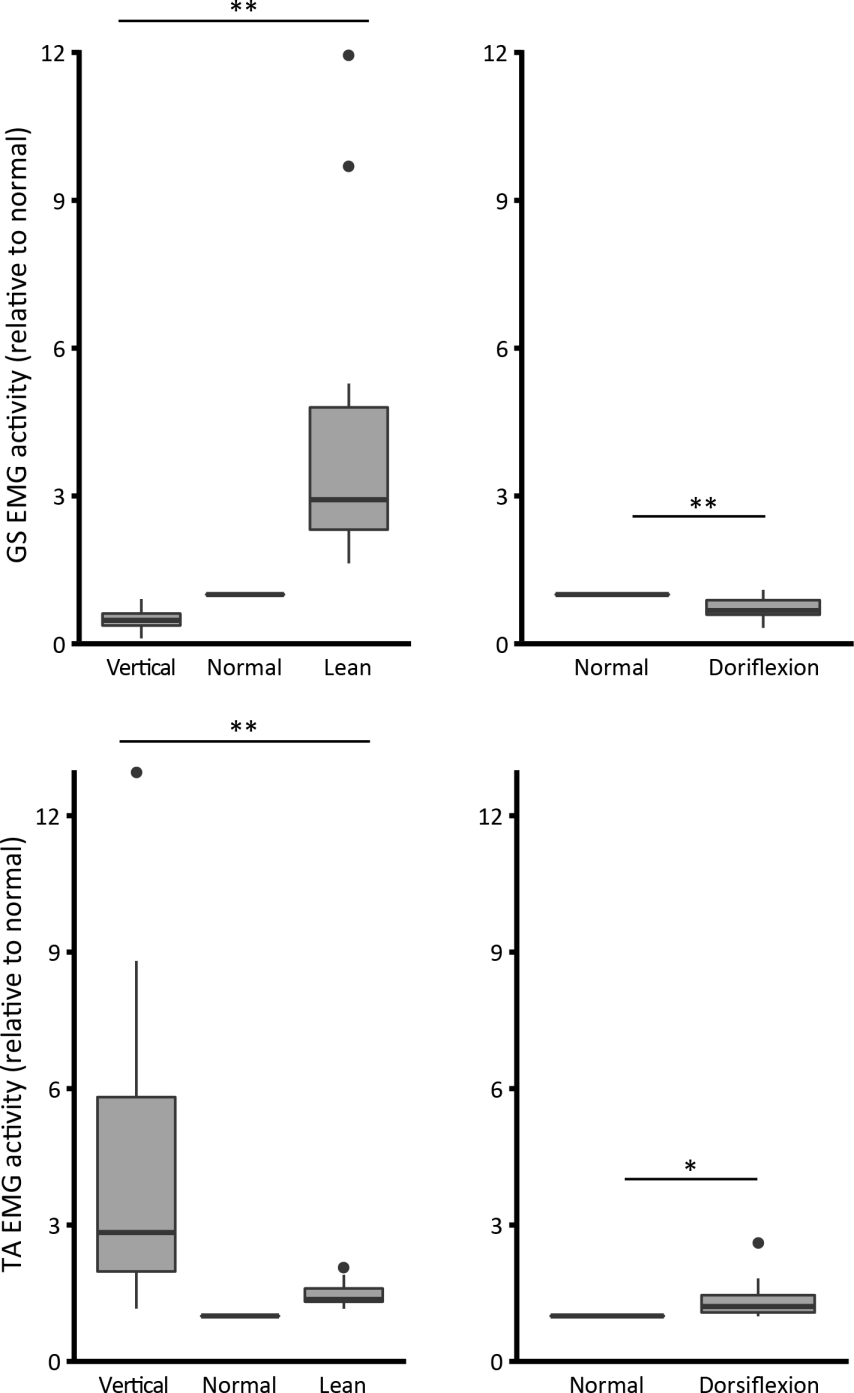
EMG activity ratio (relative to normal condition). (*) indicates significance of P<0.05, and (**) indicates P<0.001.

The analysis of EMG activity confirms that changing the level of body leaning either backwards or forwards from normal stance will alter the neural activation. Fig 7 shows normalized data. As expected, by leaning backwards (*vertical* condition), the *medial gastrocnemius* (GM) activity is reduced, but there is a large increase in *tibialis anterior* (TA) activity. When leaning forwards, there is a considerable increase in GM activity accompanied by a trivial increase in TA activity (one-way ANOVA, GM F_2,27_ = 11.7; p<0.001; TA F_2,27_ = 12.6; p<0.001) (left graph). In the dorsiflexion condition (right graph), there is a significant increase in TA activity and decrease in GM activity (GM t_(9)_ = 3.6; p = 0.006; TA t_(9)_ = -2.5; p = 0.032).

## Discussion

In this paper we attempt to understand how intrinsic ankle stiffness can be altered by changes in normal standing conditions. To complement previous research on standing individuals [2,34], two different techniques were used.

Firstly, active ankle torque was altered by asking the participants to lean at different angles. Intrinsic stiffness was shown to correlate positively with increasing levels of forward leaning which produced increasing levels of active ankle torque (increase of ~ 26.1 Nm from *vertical* to *lean*, Fig 3). These results support Casadio et al.’s [34] observation that stiffness increases with active torque in standing. This effect remained similar as we reduced the perturbation size from 0.7 to 0.1 deg. In opposition to the results presented here, for even smaller perturbations (0.05 deg), Loram & Lakie [2] have found little alteration in intrinsic stiffness with an increase in active torque.

Secondly, ankle angle was altered by rotating the standing surface by 15 deg toes-up while intrinsic stiffness was assessed. Because this caused an inadvertent postural alteration and a slight forward lean, ankle dorsiflexion increased by more than the imposed 15 deg (18.7±3.4 deg, Fig 4). We found that dorsiflexion also increased intrinsic stiffness substantially. As the participants did lean forwards a little, their ankle torque was slightly higher than normal (increase of ~ 4.8 Nm from normal to dorsiflexion, Fig 3). However, stiffness increased very considerably despite the modest torque increase. Consequently, intrinsic ankle stiffness must be increased in different ways by leaning and by dorsiflexion.

Finally, although both procedures caused significant changes in intrinsic ankle stiffness, there were only trivial alterations in the size or speed of sway. We now discuss each of these findings in turn.

### In standing, intrinsic ankle stiffness has a positive dependency on active ankle torque

The results shown here (study 1), confirm Casadio et al.’s [34] significant positive correlation between active ankle torque and standing intrinsic ankle stiffness. This relationship is clearly shown in Fig 5. For an average active ankle torque ranging from 5 to 16 to 31 Nm, the average intrinsic stiffness (mean of the three perturbation sizes) increased from 53 to 66 to 81% mgh.

Thus, a change from normal standing position to forward lean produced an average torque rise of ~ 15 Nm (Fig 3) and an average intrinsic stiffness increase of 15% mgh (Fig 5). With a mean increase in ankle torque of 13.9 Nm from normal to leaning condition, Casadio et al. [34] found an increase in intrinsic stiffness of 33.5% mgh. Thus, with slightly less increase in ankle torque, they found a larger increase in intrinsic stiffness. However, their results were derived from only two subjects, and would certainly fit comfortably into the range of values we recorded. They used a larger perturbation (ramp of 1 deg), but as we show in Fig 5, the effect of body lean upon stiffness was seen for all perturbation amplitudes.

The results from study 1 were also comparable to those previously derived from recumbent individuals [46], which also showed a significant increase in intrinsic ankle stiffness with an increase in active ankle torque. The experimental technique was very different, but the results were strikingly similar. Mirbagheri et al.’s [46] relationship between torque and stiffness is curvilinear and variable between subjects, but their Fig 6 suggests that an increase from 12 to 24 Nm would increase intrinsic stiffness by approximately 30% on average, an increase quite similar to the one reported here. Increasing stiffness with ankle torque can be simply and economically explained on the assumption that intrinsic ankle stiffness is a function of tendon stiffness. Ankle stiffness is the sum of the compliances of the structures that are deformed when the ankle is rotated. For low tensions the tendon is the weakest spring, so it dictates the upper limit of intrinsic stiffness. In effect, it is immaterial how stiff the muscles lying in series become, because the overall stiffness can never exceed that of the tendon. However, as tension increases (as in leaning forward) the tendon stiffness increases and, as a consequence, the intrinsic stiffness becomes larger.

Because the different results found by Loram & Lakie [2] and Casadio et al. [34] were possibly related to the different perturbation size used to assess stiffness, we expected to find less difference of stiffness for the smallest perturbation sizes with increase in active ankle torque. However, we did not see this interaction. The perturbation size (0.1–0.3–0.7 deg) used in study 1 was lower than in Casadio et al. [34] (1 deg), but larger than the perturbation size used by Loram & Lakie [2] (0.05 deg). In the present experiments the extreme difference of stiffness was 27.5% mgh for 0.1 deg perturbation, 22.0% for 0.3 deg and 33.1% for 0.7 deg (Fig 5, left graph). Thus there is no indication that reducing the size of the perturbation over the range that we used here causes a reduction in the stiffening associated with raised ankle torque.

Unusually, in the paper by Loram and Lakie, the authors measured both foot stiffness and ankle stiffness. They reported (see Fig 6D from Loram & Lakie [2]) that foot stiffness tended to decrease and become more consistent with increasing torque, and that this partly offset a rise in true ankle stiffness. It may be that, in using perturbations of less than a certain critical size, increasing compliance of the foot and soft tissues conceals the rise in stiffness associated with torque-induced tendon stiffening, setting a rather high and constant level of stiffness. This may be particularly relevant to quiet standing where many of the spontaneous sways tend to be very small in size. In this regimen, ankle stiffness may be effectively independent of torque level (muscle activity). Perhaps for very tiny perturbations or sways, stiffness maintains a constant level because the compliance of the foot and soft tissues acts as a relatively constant stiffness buffer. Interestingly, even when our participants were inclined as vertically as possible and applying an average ankle torque of as little as 2.2 Nm against the ground, the intrinsic stiffness was substantial, at 70% mgh when perturbed by 0.1 stimulus amplitude and 36% mgh when perturbed by 0.7 deg stimulus amplitude (Fig 5, left graph). It shows that even at conditions close to the vertical equilibrium position when there is minimal ankle torque and the ankle is as plantar flexed as far as possible commensurate within normal standing, intrinsic ankle stiffness is still relatively high.

### In standing, intrinsic ankle stiffness has a positive dependency on dorsiflexion

To our knowledge, there have not been any previous investigations into the effects of passive stretch on the intrinsic ankle stiffness of standing individuals. Here, we show that this relationship is significantly positive. With a large increase in passive stretch by 18.7 deg ankle dorsiflexion and an associated small increase in active ankle torque of ~ 4.8 Nm, stiffness increased from 50 to 77% mgh, and 77 to 109% mgh (for 0.7 and 0.1 deg perturbations, respectively) (Fig 5, right graph).

In freely standing subjects, as here, the rise in ankle torque must be associated with a forward lean and this is confirmed by Fig 4. Subjects adopted an unusual body configuration, moving the knees forward considerably and inclining the body forward a little. This arching of the body is presumably to counteract the sense of instability that arises when the toes are raised, tending to tip the body posteriorly. This rise in torque is very likely to have produced some rise in intrinsic stiffness as described above, however the increase that we observed seems much too large to be produced solely by this mechanism.

It is possible to compare our data with those from Mirbagheri et al. [46]. In that study, the authors have also shown in seated individuals that passive ankle stiffness strongly depends on passive stretch. Participants maintained a constant mean 5 Nm plantarflexion torque against the footplate and stiffness was measured in a range of angles from maximum plantarflexion to maximum dorsiflexion. When the ankle is taken from the neutral position to 14.4 deg of dorsiflexion the intrinsic stiffness increases by approximately 40% (see Fig 8 from Mirbagheri et al. [46]). Thus, these authors also report a substantial rise in intrinsic stiffness of about the same magnitude as the one we find here, and in their case, it is uncomplicated by associated torque increase. This strong relationship between ankle stiffness and passive stretch was also suggested by earlier work by Kearney and colleagues [47]. They showed that when the ankle is passively dorsiflexed from neutral until the end of the range of movement, there is relatively small change in ankle torque (ranging from 6 to 12.4 Nm plantarflexing torque), but intrinsic stiffness increases considerably (1.3 to 3.6 Nm deg^-1^).

Consequently, this increase is stiffness is associated with ankle angle rather than ankle torque so an alternative explanation for the rise in stiffness is necessary. This could be a consequence of either coactivation of antagonist muscles, increase of calf muscle moment arm, unmeasured change in knee angle acting on the biarticular gastrocnemius muscle or increased resistance of other tissues crossing the ankle.

Activity of the muscles is shown in Fig 7. In normal standing the TA muscle is virtually silent. Dorsiflexion does increase activity in the TA. However, although significant, the degree of coactivation is very slight. It can, for example, be compared to the greatly increased TA activity which is observed when the body is inclined vertically. Furthermore, in this condition of extreme dorsiflexion the TA is operating in a region of mechanical disadvantage (short fibres, reduced moment arm. Thus, the degree of coactivation seems inadequate to produce the large increase in intrinsic stiffness that we observed.

If dorsiflexion increases the moment arm of the calf muscles, a given rotation of the ankle will cause a larger linear displacement of the tendon and muscle and, consequently, measured angular stiffness will rise. However, as has been pointed out by [48–50], moment arm actually decreases with dorsiflexion. This was quantified in vivo with magnetic resonance imaging (MRI) and real-time ultrasonography by Maganaris et al. [49], who found that ankle dorsiflexion of 15 deg produces ~ 0.5 cm decrease in moment arm (Fig 2). Consequently, dorsiflexion should produce a decrease in stiffness rather than the increase that we and others observe.

In leaning forward, there might have been a slight, unintended and unmeasured extension of the knee joint. This would pull on the biarticular gastrocnemius muscle and potentially contribute to increased ankle stiffness. Similarly, during dorsiflexion the toes were moved upwards and there was an alteration in body conformation, as indicated by the sketched image in Fig 2. The shin was driven posteriorly, and this might have also acted to extend the knee slightly. Hence, this could contribute towards the increased stiffness that we measured in the dorsiflexion condition. However, the knee contribution is likely to be slight. Herbert et al. [30] showed that the relationship between gastrocnemius muscle tendon unit lengthening and joint rotation was much smaller for the knee than for the ankle. On average, ankle rotation stretches gastrocnemius by 0.83 mm per degree, whereas knee rotation stretches gastrocnemius by only 0.23 mm per degree (see Fig 1 from Herbert et al. [30]). The unmeasured knee rotation in our experiments is very unlikely to have exceeded 10 degrees. With the possibly extreme degree of extension of the knee experienced by the participants of both studies, the gastrocnemius would be lengthened by much less than 3 mm. This is very small compared to the intended lengthening produced by ankle dorsiflexion, which would be nearly 17 mm.

Given that changes in co-contraction, moment arm and knee angle do not fully explain changes in stiffness found by these studies, a remaining possibility is that the rise in stiffness is due to stretching or compression of other tissues which bridge the ankle joint.

### Tendon and aponeurosis strain as cause of increased intrinsic stiffness

The calf muscle-tendon complex can generate tension in two ways. First, with active contraction (as in the lean condition) there is a tendency for muscle to shorten and expand. This has the effect of straining the serially-arranged tendon. Series elastic structures in muscles are not limited to the extramuscular free tendon. Most muscles with a long free tendon also have a sheet-like aponeurosis that serves as a broad tendinous insertion for muscle fibers [51]. Thus, there is an associated complex biaxial straining of the aponeurosis which partly encircles the muscle [52]. On the well-established assumption that with low levels of force the tendon is less stiff than the muscle [7,53,54], the effect of the progressively increasing muscle activity required in leaning forward, is to increase tendon strain. This increases tendon stiffness and consequently ankle stiffness. Muscle stiffness is also likely to rise progressively with increased activity in leaning forward, but this is inconsequential because ankle stiffness is limited to the much lower level dictated by the tendon.

Second, the calf muscle can be passively pulled to a long length (as in the dorsiflexion experiment). This has the effect of considerably straining the aponeurosis, and because it now generates substantial passive tension at this longer length, the free tendon is similarly strained [53]. The effect of these changes is again to increase the stiffness of the tendon and consequently the ankle. In this case there is likely to be a small rise in muscle stiffness, which once again would have minimal effect on overall ankle stiffness. Direct observation of muscle/tendon motion with ultrasound would help confirm this in future studies.

Even though significant, the change in inertia in study 1 caused by condition and amplitude was very small (8.1%). Such changes are much smaller than the observed changes in stiffness, and could be accounted for simply by errors in the estimation process, since all three parameters are allowed to vary (K, B & I). For study 2, the changes were higher (14.1%), with dorsiflexion being associated with greater inertia. These apparent changes in inertia may also be due to variation in the model fitting process. Alternatively, it is concievable they are attributable to additional muscle mass being moved during the perturbation. This would be consistent with our assertion that dorsiflexion increases the stiffness of the tendon which would, in turn, cause greater muscle movement. Whether this added moving mass would be sufficient to cause the increased inertia is uncertain. Again, measurement with ultrasound would help confirm this.

### Sway size changes little in response to altered intrinsic stiffness

In a previous paper [5], we showed that ankle intrinsic stiffness is reduced when there is increased movement amplitude (more sway). Because this reduction in intrinsic stiffness was seen best with small perturbations, we attributed it to a movement induced reduction in muscle stiffness (thixotropy). Large sways are associated with low muscle stiffness and consequently low intrinsic ankle stiffness. We suggested that the converse might be true–small sways might be associated with high muscle stiffness and higher intrinsic ankle stiffness. That suggestion is not strongly supported by the present findings which show no clear relationship between ankle intrinsic stiffness and sway size (Fig 6). It seems inevitable that the tendon stiffness sets an upper limit on ankle stiffness. Because of the series arrangement of muscle and tendon, and the fact that at modest levels of torque the tendon is much less stiff than the muscle, the effect of muscle stiffness change is highly asymmetric. Movement can easily decrease muscle (and hence ankle) stiffness. Lack of movement might, indeed, increase muscle stiffness considerably but this does not increase intrinsic ankle stiffness which is limited to the value set by the tendon.

Intrinsic stiffness is a stabilizing feature because in partly offsetting gravitational acceleration it reduces the size and the speed of necessary neural interventions. However, the changes that we produced did not systematically alter sway size or velocity. It is possible that by requiring subjects to stand in unusual configurations, as here, the normally stabilizing role of intrinsic stiffness is vitiated by the unfamiliarity of the task and standing is dominated by active neural control so that any departure from normal tends to increase sway size.

### Contributions to the ankle torque required to stand

Because both processes stretch elastic tendon structures, muscle activity and dorsiflexion can, in principle, contribute to the ankle torque required to stand. Weiss et al. [47,55] showed that with large passive dorsiflexion their subjects generated an ankle torque ranging from 6 to 12.4 Nm. They suggested that as normal standing requires a total torque of the order of ~ 50 Nm the passive torque from both ankles might make a material contribution. We confirm this suggestion. We show that, with extreme dorsiflexion, muscle activity in standing can be decreased, even though ankle torque demand is higher because dorsiflexion causes an inadvertent slight extra lean forward. However, with the very small degree of dorsiflexion that occurs in standing on level ground the passive torque contribution to normal standing is likely to be very small. Our conclusion is that in normal standing ankle stiffness will be much more affected by modulation of ankle torque (which varies a lot) rather than ankle angle (which varies only a little).

## Conclusion

Our results show that intrinsic ankle stiffness in standing individuals increases considerably in conditions of increased forward lean and dorsiflexion. Even though stiffness is changed, body sway is little affected. Since during normal standing there is relatively small potential for changes in ankle angle compared with larges changes in ankle torque, we believe that intrinsic ankle stiffness is mostly affected by the latter.
